# A hybrid multi-node QKD-ECC architecture for securing IoT networks

**DOI:** 10.1038/s41598-025-17184-x

**Published:** 2025-09-25

**Authors:** Rajnish Chaturvedi, Dinesh Sahu, Brijendra Pratap Singh, Shiv Prakash, Tiansheng Yang, Rajkumar Singh Rathore, Korhan Cengiz, Nikola Ivković

**Affiliations:** 1 SCSET, Bennett University, Greater Noida, Uttar Pradesh 201310 India; 2 Department of Electronics and Communication, University of Allahabad, Prayag Raj, Uttar Pradesh India; 3 University of South Wales Pontypridd, Rhondda Cynon Taf, United Kingdom; 4 Cardiff School of Technologies, Cardiff Metropolitan University, Cardiff, United Kingdom; 5 Department of Electrical Engineering, Prince Mohammad Bin Fahd University, 31952 Al Khobar, Saudi Arabia; 6 Faculty of Organization and Informatics, University of Zagreb, Pavlinska 2, 42000 Varaždin, Croatia

**Keywords:** Quantum key distribution (QKD), Elliptic curve cryptography (ECC), IoT network security, Multi-node communication, Quantum cryptography, Computer science, Information technology

## Abstract

The rapid expansion of Internet of Things (IoT) applications in sectors like smart cities, healthcare, and industrial automation has introduced serious security challenges due to limited device resources and growing threats from quantum computing. Traditional cryptographic techniques such as RSA and AES are increasingly inadequate, particularly against quantum attacks, and face limitations in scalability and efficiency in multi-node environments. To overcome these challenges, this paper proposes a lightweight and quantum-resilient security framework for IoT networks based on Multi-Node Quantum Key Distribution (QKD) integrated with Elliptic Curve Cryptography (ECC), termed MNQ-ECC. The proposed architecture enables secure key generation and exchange across multiple nodes and includes four security phases: pre-deployment, registration, login, and authentication. Here, performance evaluation is carried out using Qiskit simulators under varying network conditions and key performance metrics such as key generation rate, entropy, latency, and communication overhead are analysed. The results demonstrate that MNQ-ECC achieves 99.5% resistance to quantum attacks, improves key generation efficiency by 30%, and reduces encryption overhead by 20% compared to standard ECC. These findings confirm the framework’s effectiveness in securing IoT networks with high scalability, low latency, and strong resilience against classical and quantum threats.

## Introduction

**Fig. 1:**
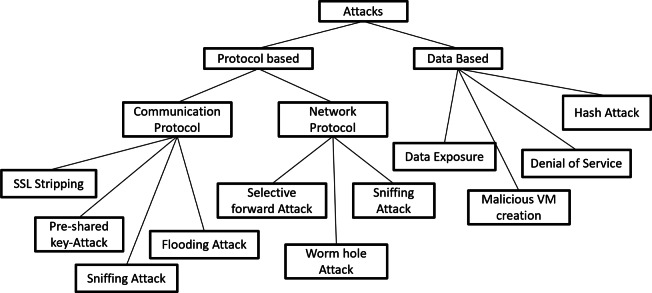
IoT attacks classification.

Nowadays, computer technology is evolving every day and one of the best evolutions is toward automation. In this era, we are trying to automate everything like industry, education, home appliances, vehicles, agriculture processes, etc. To automate everything, people design various physical devices that have sensors to take raw data from the environment, use the internet for connection, communication, and data processing, and actuators for acting accordingly. These devices are called the Internet of Things (IoT) and communicate between transient states by using embedded software. At the beginning of the automation era, we had a small IoT network where physical devices were connected through internet. Now, due to increase in demand for IoT devices, The scale of the internet of things has extended to from the local workstation to Industrial IoT frameworks. Widespread of IoT devices and the huge transmission of data among IoT devices and servers increase the risk of data accessing, manipulation, or deletion. Another issue with IoT is many IoT devices are remotely operated by users so if there is no proper authentication between a valid user and IoT device, an attacker can easily access the IoT device and can disable its functionality or misuse it. Therefore, security is a major challenge in IoT networks to save data from attackers. In IoT networks, various attacks are possible due to vulnerability in the network^1^ such as Flooding attacks, pre-shared key attacks, sniffing attack, wormhole attacks, sniffing attacks, hash attacks, Botnets, DDoS, Ransomware, AI-based attacks, Eavesdropping attacks, Privilege escalation attack, brute force attack, etc. Attacker intends to interrupt the services or access the information by performing any of these attacks. Here, the author classifies these attacks into two broad categories based on their attacking nature: protocol-based and Data based as demonstrated in Fig. 1. Protocol-based attacks are further categorized into two subcategories: communication protocol and network protocol.

**Fig. 2:**
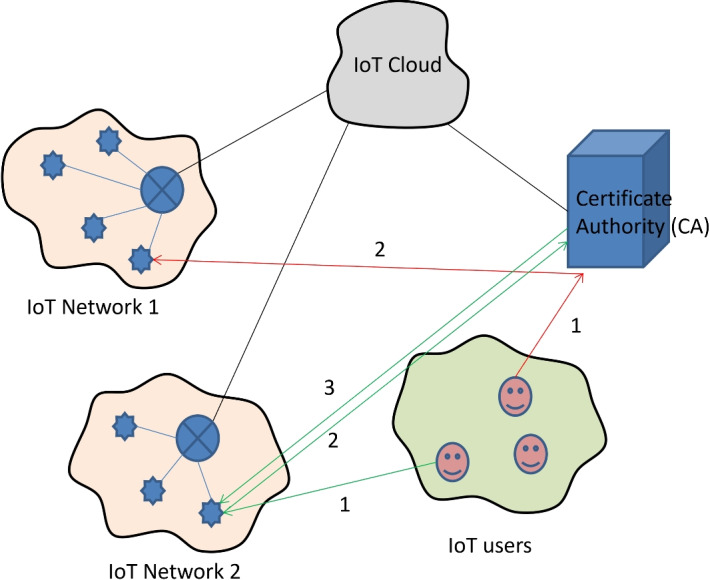
Connectivity among IoT devices, servers, and IoT users.

Most IoT devices are small in size and lightweight and have limited memory, low energy, and low processing power. Therefore, data storage and processing of data are done in the cloud in IoT networks. These devices are controlled or accessed by a user remotely either through the IoT cloud using internet or directly (peer to peer) as demonstrated in Fig. 2. An attacker takes the benefit of this to perform an attack either by accessing the IoT cloud or getting access to the IoT device ^30,31,32^.

To protect the data and services in IoT networks from attackers, a good security mechanism plays a very important role. Many researchers have proposed various security mechanisms^2–6^ but still IoT network has some vulnerability. As we know that existing cryptographic techniques, such as RSA and AES, are vulnerable to quantum computing attacks, making IoT networks susceptible to breaches. Furthermore, Due to limited resources in IoT require lightweight yet highly secure encryption mechanisms. A robust security solution is needed that not only ensures data confidentiality and integrity but also provides quantum resistance while maintaining computational efficiency. Several researchers have attempted to address IoT security through encryption techniques, authentication protocols, and trust management frameworks. Traditional security models fail to offer resilience against quantum-based attacks. Some quantum cryptography-based security mechanisms have been proposed, including point-to-point Quantum Key Distribution (QKD), but they face scalability issues in multi-node IoT networks. Moreover, existing security solutions often overlook efficient session key management and authentication across distributed IoT environments. Here author analyzes the existing IoT security mechanisms and tries to find out existing issues. Based on existing issues, the author proposes a novel security mechanism based on Quantum Key Distribution (QKD) and Elliptic Curve Cryptography (ECC). There are various security techniques based on Quantum Key Distribution (QKD) like BB84 Protocol Implementation, Integration of QKD Modules in IoT Devices, Quantum Key Distribution as a Service (QKDaaS), Key Reconciliation and Privacy Amplification Techniques, QKD-enabled Secure Bootstrapping, Entanglement-based QKD Schemes, etc. Out of these techniques, one more popular QKD technique known as Multi-Node QKD Networks, provides better security compared to other authentication mechanisms and devices consume less power as compared to others. Here author proposes a security mechanism for IoT networks by using Multi-Node QKD Networks and ECC. Traditional QKD schemes^7^ typically involve point-to-point key distribution between two parties. However, in IoT networks, multiple devices often need to communicate securely with each other, requiring a more complex network topology. Multi-Node QKD Network^8,34^ extends QKD capabilities to support secure communication among multiple nodes within the IoT network. Its duties are as follows:

1. Network topology: A multi-node QKD network involves in connecting multiple nodes collectively. This topology can be mesh, star, or some other suitable configuration relying on the IoT utility necessities.
2. Multi-node key distribution: Multi-node QKD generates common keys among pairs of nodes inside the network. that is done via technology like quantum repeaters or quantum relay nodes, which help to distribute qubits throughout the network.
3. key control and sharing: once the entangled qubits are distributed throughout the network, every node shares key material with its neighboring nodes through entanglement swapping or different quantum operations. This permits the establishment of pairwise secure quantum keys between adjoining nodes, making sure end-to-end protection for communication in the network.
4. Dynamic key renewal: In a dynamic IoT environment wherein nodes can often be a part of or leave the network, QKD network provides the dynamic key renewal mechanism. while new nodes be a part of the network or present nodes depart, the QKD network dynamically adjusts the key distribution to make sure the security and integrity of communications.
5. Resilience and fault tolerance: Multi-Node QKD networks are designed to be resilient to faults and attacks. By way of using redundancy and errors correction strategies, Multi-Node QKD networks save networks failure or eavesdropping without compromising the security of the distributed keys.
6. Scalability and efficiency: Multi-node QKD networks scale to accommodate large-scale IoT deployment with massive numbers of interconnected devices. with the aid of optimizing the allocation of resources and communication protocols, Multi-node QKD networks allow efficient deployment while minimizing overhead and latency.
7. Integration with classical cryptography: While QKD provides security without compromising key distribution, it can be combined with classical cryptography for better security. Multi-node QKD networks can be combined with classical encryption algorithms such as AES or ECC to provide quantum-secure key distribution and strong data encryption

Such unprecedented levels of security and privacy which ensure secure communication among connected devices even under sophisticated attacks on IoTs can be protected by adopting IoT deployments based on Multi-Node QKD with ECC mechanism. This is indeed a major milestone towards securing internet-of-things networks using quantum power.

### Motivation and contribution

The rapid proliferation of IoT devices across domains such as smart cities, healthcare, and industrial automation has intensified the need for secure, efficient, and scalable communication frameworks. Existing security protocols often fall short in protecting against sophisticated threats, especially those posed by quantum computing. Additionally, resource-constrained IoT devices require lightweight encryption mechanisms that traditional post-quantum cryptographic techniques often fail to support due to their large key sizes or high computational overhead. In the past, Point-to-point QKD solutions have been explored to handle these issues, but they lack scalability for dynamic multi-node environments.

To address these challenges, this paper proposes a novel Multi-Node QKD integrated with Elliptic Curve Cryptography (MNQ-ECC) framework for IoT networks. The key contributions of the work are:

1. A secure and scalable architecture for IoT networks that integrates Multi-Node QKD for quantum-resilient key generation and ECC for lightweight encryption.
2. A four-phase authentication model including pre-deployment, registration, login, and authentication phases to ensure end-to-end secure key generation and session management.
3. Mathematical modeling and algorithms for each phase of the framework, facilitating rigorous implementation and simulation.
4. Security analysis showing resilience against a wide range of attacks including eavesdropping, man-in-the-middle, brute-force, replay, key compromise, and quantum attacks.
5. Performance evaluation through simulation using tools like Qiskit, demonstrating the framework’s scalability, low latency, and high entropy in session key generation.
6. Comparative study with existing security protocols, highlighting superior performance in terms of key generation speed, attack resilience, and communication overhead.

This work bridges the gap between practical IoT deployment requirements and the theoretical advantages of quantum cryptography by combining them into a cohesive and implementable framework.

The rest of the paper is organized as follows: Section 2 defines the literature on IoT security mechanisms and tries to find out possible existing issues. In section 3, the Author proposes an authentication framework that helps to design a robust security mechanism. Section 4 proposes a new robust security mechanism for IoT networks. The Analysis of the security level of the proposed mechanism is detailed in Section 5. Section 6 details the performance measurement of the proposed method. Section 7 details the type of attacks that can be prevented by the proposed method. Section 8 concludes the complete work and section 9 details the future direction.

## Literature review

**Fig. 3:**
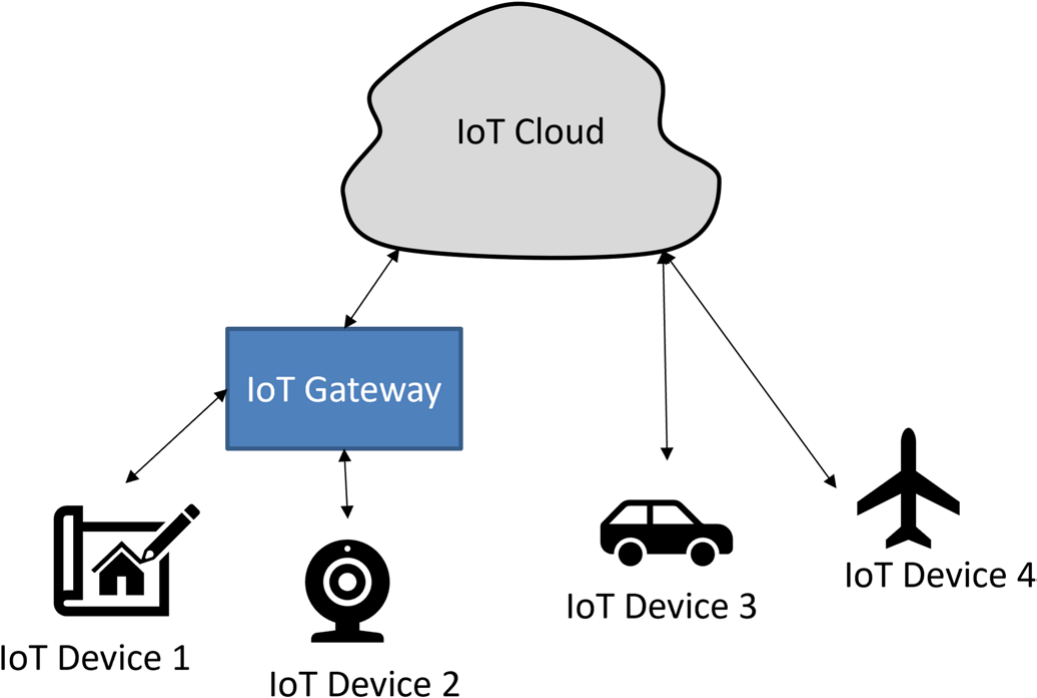
Mode of communication between IoT devices and IoT cloud.

Today most industries are moving towards automation. IoT plays a very important role in automation and generates huge digital data from every field. Since most of the IoT devices are lightweight and low-energy devices, therefore these IoT devices are equipped with the cloud for data processing. These devices communicate with the server placed into the cloud either directly or through an IoT gateway as shown in Fig. 3. We have seen a major boom in the IoT commercial sector in the last few years. The internet of things includes smart vehicles, smart home appliances, and smart labs. It’s easy to live but too much dependability can lead to high risk. Since IoT devices are lightweight, use lightweight protocols. So the current trends for intrusion activity by attackers are IoT networks^9^.

IoT device security is a crucial aspect of the modern connected world. With the increasing number of IoT devices and the ability to collect, store, and transmit sensitive data, the risk of cyber-attacks, privacy violations, and data breaches rises. we need to understand the common security weaknesses of IoT devices, such as outdated software, lack of encryption, weak passwords, and the ability to be easily hacked. Consider the type of threats to IoT devices, which include malware, DDoS assaults, and unauthorized entry to get sensitive information. To provide an explanation for the importance of IoT device security, some real-life instances of an IoT safety incident and its consequences are given here. They help highlight the significance of IoT protection and the outcomes of ignoring it. The Mirai botnet attack is a massive DDoS attack that was launched in 2016, referred to as Mirai^10^, that took down numerous popular websites. The botnet includes thousands of unsecured IoT gadgets, including cameras and routers. The target data breach In 2013, a data breach at Target affected 40 million bank credit and debit cards^11^. The problem was determined to be caused by the vendor’s HVAC system connecting to the internet without security measures. St. Jude Medical Implantable Heart Device Attack. In 2016, a security researcher in St. Jude Medical’s finds out that an implantable heart device can be attacked and controlled by remote hackers^12^. The discovery led to a device recall and increased scrutiny of medical device safety. In 2015, security researchers discovered that they could control the Jeep Cherokee via an internet connection^13^. The incident highlights the risks posed by connected vehicles and the need for the automotive industry to improve security^33^

These case studies tell us that IoT security should be taken seriously, and ignoring it can lead to serious consequences. As we have seen, different types of attacks are possible in IoT networks based on the nature of the attacks. In this work, the author mainly focuses on the security of key sharing, login, and authentication processes. Various researchers have worked in the security of IoT networks^14–21^ and found various vulnerabilities. Here, we discuss a few existing security mechanisms for IoT networks and their vulnerabilities. The authors in^14^ proposed a certificate-based pairwise key establishment symmetric key protocol for wireless sensor networks. Here authors used elliptic curve cryptography to generate a symmetric key between two parties. In this approach, the authors used a third-party server (certificate authority) to verify the valid users. However, if attackers compromise the security of a third-party server, it can allow malicious users to exchange the secret with a valid user and can easily get the data. In the paper^15^, the authors presented a lightweight and secure session key establishment mechanism for smart homes and claimed that the proposed algorithm provides authenticity, confidentiality, and other security goals. However, there are few potential security concerns like authors presumed home gateway is a tamper proof device, lack of key management, scalability issue, reliance on third party, replay attack and forward secrecy. The authors in^16^ proposed a password-based user authentication scheme for large-scale hierarchical WSN. This scheme uses a smart card to store the password and allows dynamic node join. However, authors in^17^ proved that the dynamic password-based authentication scheme^16^ is not suitable for realistic applications. Here, authors in^17^ identified some redundant factors in the dynamic password-based authentication scheme that slow down it and to enhance the performance of this scheme, they proposed a new enhanced dynamic password-based authentication scheme. Paper^18^ proposed a lightweight temporal credential-based mutual authentication model for WSN. Here, five different authentication schemes are designed to achieve authentication among user sensors and cloud gateway according to different steps in different models. However, in the next year, authors in^19^ found some performance-affecting factors in the authentication scheme in^18^ that degraded the performance of the authentication scheme and proposed a new scheme. Authors in^20^ claimed that Turkanovic et al. (2014) based scheme still has some performance issues and proposed an efficient user authentication and key agreements scheme for heterogeneous wireless sensor networks. However, Xin et al. (2018)^21^ stated that the proposed scheme in^20^ is still insecure and it fails to resist passwords through password guessing attack. To safeguard the WSN from potential attacks, Xin et al. (2018) suggested a new authentication scheme that operates in two levels: authentication and login. The scope of this study is limited to safeguarding lightweight sensor devices from known network layer and physical layer-based attacks. For detecting malicious nodes in IoT networks, Alsheri and Hussain^2^ proposed a cluster-based fuzzy logic model where existing nodes are grouped into clusters. Here authors propoesd trust management in IoT by proposing two methods. In the first method, authors claimed to detect contradictory attacks, on-off attacks, and other malicious nodes. In the second method, the authors claimed a secure message system for IoT nodes. However, the proposed methods do not cover the data audit attack. As quantum computers advance, researchers have shifted towards Post-Quantum Cryptography (PQC), which includes classical encryption techniques resistant to quantum attacks. Some of the leading PQC approaches for IoT security include: Lattice-Based Cryptography in which Algorithms like NTRUEncrypt^22^ and Kyber^23^ provide quantum-resistant encryption with relatively low computational cost, making them suitable for IoT. Code-Based Cryptography includes Techniques such as McEliece^24^ encryption use error-correcting codes for security, but they require large key sizes, making implementation in IoT challenging. Multivariate Polynomial Cryptography methods, like Rainbow signatures^25, 35^, offer strong security but require optimization for resource-limited IoT environments. Hash-Based Cryptography includes Signature schemes like SPHINCS+^26^ provide quantum security and are being considered for lightweight authentication in IoT. While PQC offers quantum-resistant security, it still faces implementation challenges in IoT networks, such as key size overhead, computational complexity, and compatibility with existing cryptographic infrastructure. Table 1 summarizes key existing IoT security approaches, their core contributions, and the associated limitations. This comparative analysis of the literature helps to identify the gaps that we aim to address in our proposed work, particularly in the domains of secure key management, scalability, quantum-resistance, and resource efficiency in IoT networks.

**Table 1:** Comparison of Cryptographic Protocols in IoT Authentication.

Ref	Method / Protocol	Core Technique Used	Key Features	Identified Limitations
^14^	Certificate-based Pairwise Key Establishment	ECC with third-party CA	Lightweight symmetric key establishment	Vulnerable if CA is compromised
^15^	Lightweight Session Key Establishment	Symmetric encryption with gateway	Supports smart homes	Assumes tamper-proof gateway; lacks key management
^16^	Password-based Authentication	Smart card + password	Dynamic node join	Performance degradation; security concerns
^17^	Enhanced Password-based Scheme	Optimized dynamic password approach	Improved over^16^	Redundant operations affect efficiency
^18^	Temporal Credential Mutual Authentication	Five-phase model with cloud gateway	Supports multi-step auth	Later proven inefficient by^19^
^19^	Enhanced Mutual Authentication	Lightweight ECC + timestamps	Improved performance	Still lacked quantum resistance
^20^	Authentication for Heterogeneous WSN	ECC + session key agreement	Tailored for IoT	Insecure against password guessing (as per^21^)
^21^	Anonymous Authentication for WSN	ECC + dual-level authentication	Improved security levels	Focused only on network/physical layers
^2^	Cluster-based Fuzzy Trust Model	Trust scoring for clusters	Detects on-off and contradictory attacks	Doesn’t cover data audit threats
^22–26^	Post-Quantum Cryptography (e.g., NTRU, Kyber, SPHINCS+)	Lattice, Code, Hash-based	Quantum-resistant algorithms	High key size, resource heavy for IoT

As we have seen in the literature, many researchers have proposed various security methods to protect IoT devices from different attacks, but still, we are lacking some security concerns in IoT networks like lightweight security mechanisms, secure session key generation, and secure the IoT networks from various attacks as shown in Table 1. So, in this paper, the author proposes a new lightweight secure security mechanism that tries to protect IoT networks from various attacks. In the next section author proposes a security framework that helps to design a robust security mechanism.

## Proposed authentication model

**Fig. 4:**
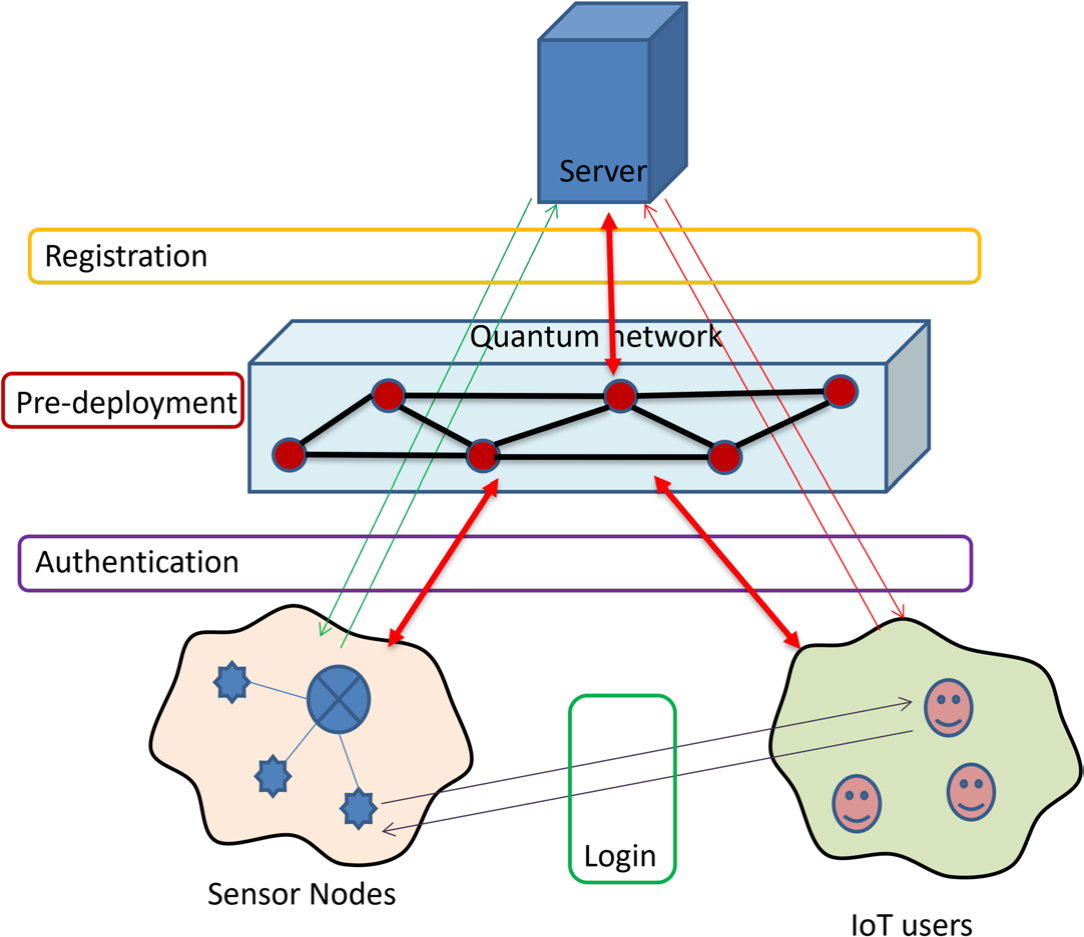
Mode of communication between IoT devices and IoT cloud.

To design a robust security mechanism, there should be a good security framework to generate the secure key in between authenticated bodies. According to IoT infrastructure, an attacker can perform the attack in different ways as by attacking sensor nodes, servers, user devices, communication channel in between sensor node and server, communication channel in between user and server, communication channel in between sensor node and user. Due to these vulnerability in the IoT networks, an attacker can perform the attacks and do malicious things. To secure the IoT network, pair of nodes in the networks should have to share or generate the secure key for further communication. This can be achieved authentication and the authentication process can be done in different ways like authors in paper^21^ used two models for authentication, authors in papers^3,14^ used a simple authentication process. Here Author proposes a security framework for secure key generation and authentication as depicted in Fig. 4. The proposed system runs through four stages to set a secure key between authenticated nodes of an IoT network. First, a particular network topology (star, ring, or mesh) is used to deploy the server, quantum networks, clusters of sensor nodes, and users during the pre-deployment phase, and their identities are known to the server. During the registration phase, the server stores the identity of both users and IoTs and creates cryptographic keys. This is followed by the process of login, during which the user tries to get connected with sensor nodes and start developing a secure key session. Lastly, at the authentication stage, the user confirms the information exchange together with the sensor nodes via the server. This is a systematic, organized method that improves the authentication process, in this manner, making IoT networks resistant to different attacks and reducing the number of vulnerabilities .

To protect IoT networks from attackers, the author identifies the possible goals for proposed security mechanism that need to be satisfied. The proposed model suggests following security goals based on four different phases (pre-deployment phase, registration phase, login phase, and authentication phase) to generate secure pairwise key between authenticated parties: secure IoT networks from attackers in the pre-deployment phase; secure IoT networks from attackers in the registration phase; secure IoT networks from attackers in the login phase; secure IoT networks from attackers in the authentication phase; provide forward and backward secrecy. In the next section, this paper proposes a security method that fulfill the above-defined goals in order to protect IoT networks from various types of attacks.

## Proposed method

As seen previous work, the IoT networks have various security issues like different attacks, latency, computation power, etc. To overcome these issues, here author proposes a security mechanism by using a novel mechanism based on Quantum Key Distribution (QKD): Multi-Node QKD Networks, with Elliptic Curve Cryptography (ECC). The proposed mechanism is designed in such a way that it can protect the IoT networks from attackers in different security phases to secure IoT networks from attackers including the pre-deployment phase, attackers in the registration phase, attackers in the login phase, attackers in the authentication phase and provide forward and backward secrecy By adopting Multi-Node QKD Networks, IoT deployments can achieve unprecedented levels of security and privacy, ensuring that communication among interconnected devices remains secure even in the face of sophisticated eavesdropping attacks. This approach represents a significant advance in harnessing the power of quantum technology to secure IoT networks. Let’s take a closer look on few important aspects of the Quantum Key Distribution (QKD) network that are used in proposed security mechanism: Two neighboring nodes in the network establishes the quantum entanglement. Mathematically, consider an entangled state with multiple qubits shared between (N) nodes, expressed as:

1 $$\begin{aligned} {|{\Psi _{\text {ent}}}\rangle } = \frac{1}{\sqrt{2}} \sum _{i=0}^{2^N - 1} {|{i}\rangle }_1 {|{i}\rangle }_2 \cdots {|{i}\rangle }_N \end{aligned}$$

where ${|{i}\rangle }_k$ represents the state of the $k$-th qubit. Once entanglement is established, a quantum key distribution protocol (such as BB84 or E91) can be used for key distribution. Entangled qubit of each node (i) is shared with its neighbor (j). Mathematically, the state of the entangled qubits shared between nodes $i$ and $j$ can be represented as:

2 $$\begin{aligned} {|{\Psi _{ij}}\rangle } = \frac{1}{\sqrt{2}} \left( {|{00}\rangle } + {|{11}\rangle } \right) \end{aligned}$$

for a maximally entangled Bell state.

Nodes measure their respective qubits and exchange the measured results. The node generates a secure key based on the measured results and shared state. Mathematically, the raw key bits $k_{raw}$ are obtained through a basis reconciliation process:

3 $$\begin{aligned} k_{raw} = (m_i== m_j ) \end{aligned}$$

where $m_i$ and $m_j$ are the measurement results of nodes i and j, respectively.

The quantum key generated by QKD can be easily integrated with classical encryption algorithms. Classical encryption algorithms such as AES or ECC with the quantum-generated keys providing a secure foundation for key distribution and for data encryption.

**Fig. 5:**
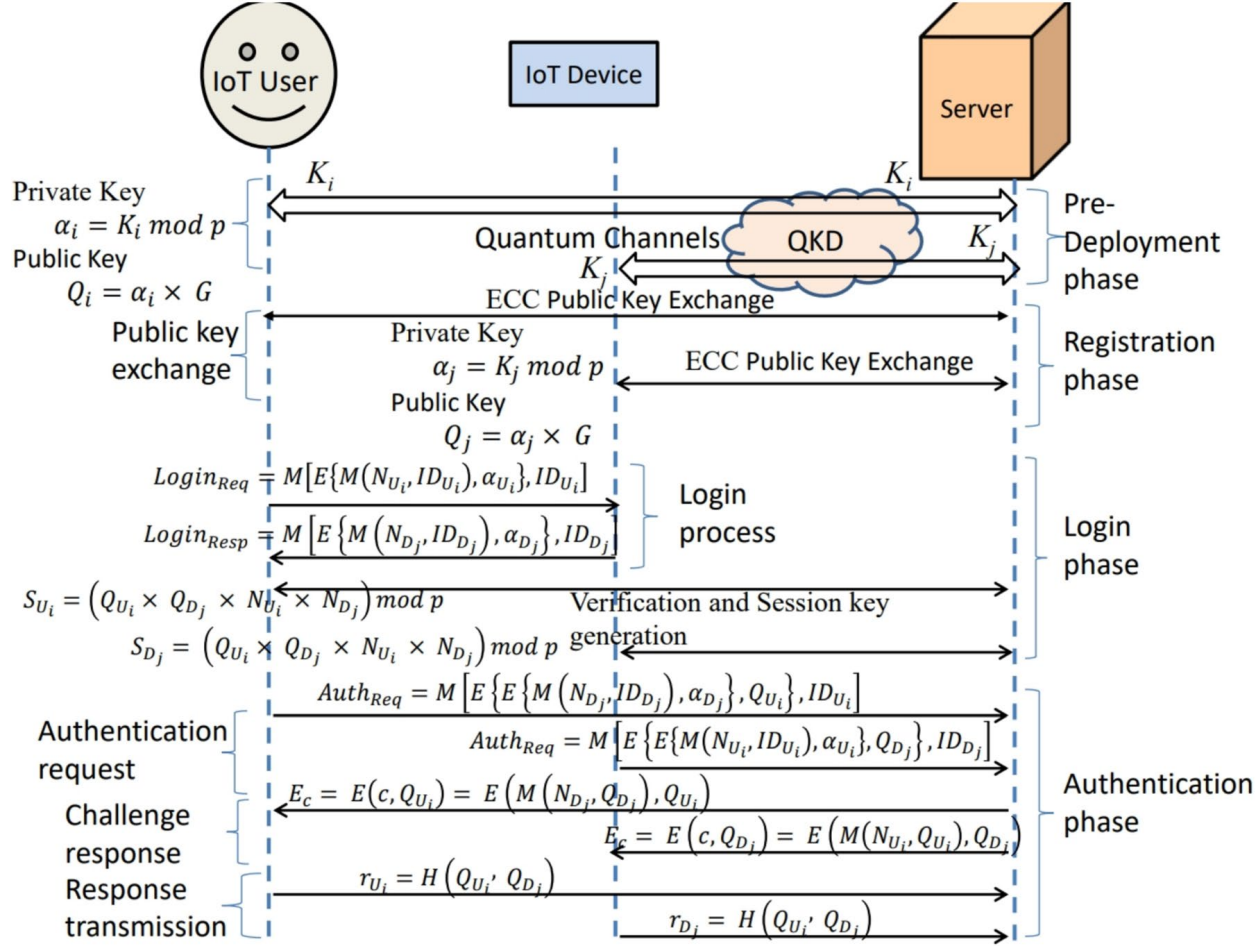
Overall flow diagram of proposed MNQ-ECC for IoT network.

Here, author suggests a security technique that uses multiple quantum key distribution nodes (QKD) and elliptic curve cryptography (ECC), called multi-node QKD with ECC (MNQ-ECC), to offers robust security framework to ensure protection of IoT networks from various attacks. It ensures the secure key generation, key distribution, and data encryption to protect against attackers and unauthorized access as depicted in Fig. 5. The proposed mechanism is divided into four sub-algorithms according to different security phases of proposed authentication model to achieve high security. The symbols used in proposed MNQ-ECC are defined in Table 2.

**Table 2:** Symbols and their description used in proposed MNQ-ECC.

Symbol	Description
${|{\Psi }\rangle }$	Quantum state
${|{0}\rangle }, {|{1}\rangle }$	Qubit states
$K_i$	Quantum key of *i*-th node/user
*p*	Prime modulus
$E(\mathbb {F}_p)$	Elliptic curve parameters
$\alpha _i$	Private key
$Q_i$	Public key
*G*	Elliptic curve base point
$ID_i$	User/device ID
$N_U, N_D$	User and device Nonces
*M*(*x*)	Message containing information *x*
*E*(*x*, *y*)	Encryption of *x* using key *y*
*D*(*x*, *y*)	Decryption of *x* using key *y*
$S_U, S_D$	User and device session keys
*H*(*x*, *y*)	Hash function

In the pre-deployment section, MNQ-ECC is used to provide the safety of IoT networks with the aid of establishing secure communications. In this section, MNQ-ECC makes use of Multi-Node QKD technology to generate quantum keys for customers, IoT gadgets, and servers. The user uses those keys to establish secure communication between the IoT devices and the server. algorithm 1 details the steps concerned inside the pre-deployment phase of MNQ-ECC.

**Algorithm 1:**
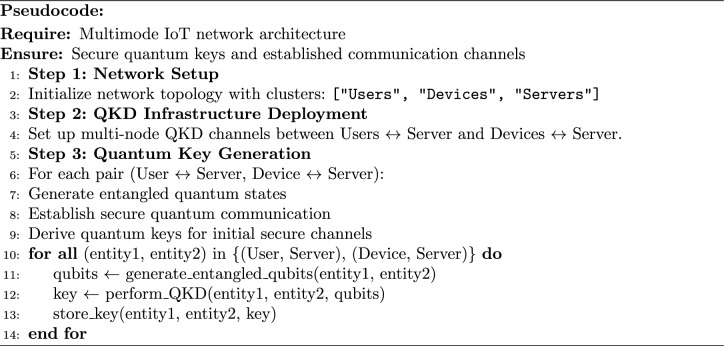
MNQ-ECC in the Pre-Deployment Phase

In the pre-deployment section of the authentication mechanism using MNQ-ECC in a multimode IoT network, numerous steps to be taken to set up the network infrastructure and generate quantum keys securely as described in algorithm 1. Below are the detailed steps of algorithm 1along with mathematical descriptions:

1. **Network Setup:** determine the topology of the IoT network, consisting of the association of clusters (customers, IoT gadgets, and servers) and the communication links between them.
2. **QKD Infrastructure Setup:** Configure the QKD infrastructure to support multimode communication. This involves setting up QKD nodes and establishing quantum communication channels between them.
3. **Quantum key generation:** Prior to deploying IoT devices, Multi-Node QKD establishes secure cryptographic keys among network nodes. Multi-Node QKD generates cryptographic keys based on the principles of quantum mechanics. Let’s denote the quantum states generated by QKD as ${|{\Psi }\rangle }$ Mathematically, the quantum states can be represented as:
  4 $$\begin{aligned} {|{\Psi }\rangle } = \sum _i \left( \alpha _i {|{0}\rangle }_i {|{0}\rangle }'_i + \beta _i {|{1}\rangle }_i {|{1}\rangle }'_i \right) \end{aligned}$$
  where ${|{0}\rangle }_i$ and ${|{1}\rangle }_i$ represent the qubit states at node $i$, and ${|{0}\rangle }'_i$ and ${|{1}\rangle }'_i$ represent the qubit states at the neighboring node $i'$. Utilize Multi-Node QKD to establish secure communication channels between each pair of device/user and the server. This involves securely generating quantum keys over the quantum communication network. Let’s denote the generated quantum keys as $K_i$ at $i^{th}$ node. Once the quantum keys are generated, classical communication channels are established using the shared secret keys derived from the QKD process.

These steps provide secure communication and authentication in IoT networks in the pre-deployment phase. Protect IoT networks from attackers during registration using MNQ-ECC, which is involved in establishing secure communication between network infrastructure and IoT devices/users to ensure the integrity and confidentiality of sensitive information. This is achieved by generating and sharing the ECC keys between the server and user, and server and IoT device. To achieve this author uses MNQ-ECC as described in Algorithm 2:

**Algorithm 2:**
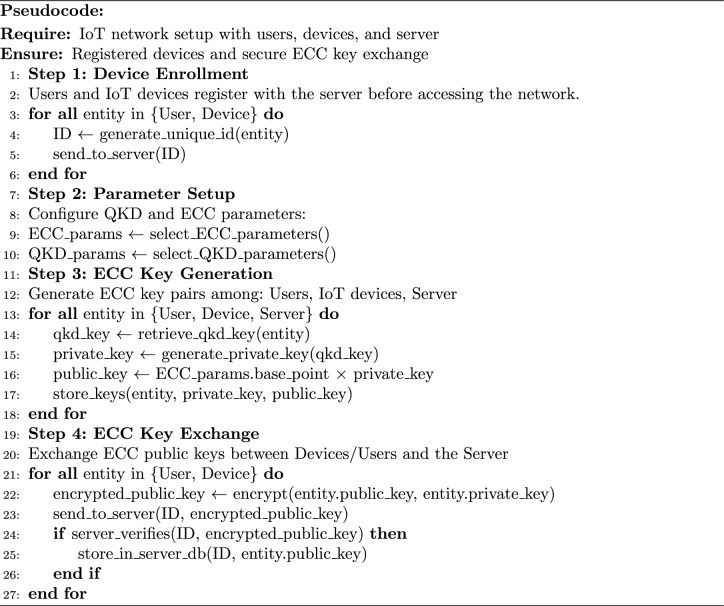
MNQ-ECC in the Registration Phase

Real-time registration of authentication technology using quantum key distribution (QKD) and elliptic curve cryptography (ECC) in various IoT networks to secure the registration of users and IoT devices agent to the network and generate an encryption and decryption keys used for secure communication: the detail steps of Algorithm 2 are defined here:

1. **Device Enrollment:** Users and IoT devices must register with the server before accessing the network. During registration, each device is assigned a unique identification number (ID) and is associated with the public ECC number.
2. Parameter Setup:
  QKD Parameters: Choose parameters for the QKD protocol, such as the quantum signal modulation, detection basis, error correction, and privacy amplification schemes.
  Elliptic Curve Parameters Selection: Choose appropriate elliptic curve parameters (e.g., curve equation, base point, prime modulus) for ECC key generation. Let’s denote the elliptic curve parameters as E($F_p$ ), where p is the prime modulus.
3. **ECC Key Generation:** For each entity (users, IoT devices, servers), generate an ECC key pair ($\alpha$,Q), where $\alpha$ is the private key, and Q is the corresponding public key represented as an elliptic curve point. Mathematically, the ECC key pair generation involves: Use the generated quantum key $K_i$ as a random number to generate the private key. Compute the private key:
  $$\begin{aligned} \alpha _i = K_i \mod p \end{aligned}$$
  Compute the public key:
  $$\begin{aligned} Q_i = \alpha _i \times G \end{aligned}$$
  where $G$ is the base point of the elliptic curve.
4. **ECC Key Exchange:** The device/user exchanges the ECC public key with the server for future secure communication using ECC-based encryption algorithms.
  - **Public Key Transmission:** Each secure device/user sends its public key $Q_i$ to the server. This transmission is done via an encrypted mode (encryption using the private key) along with respective device/user identifiers ($ID_i$) to ensure confidentiality and integrity.
  - **Public Keys Reception by the Server:** After transmission, devices/users’ public keys $Q_i$ are securely received by the server. The server decrypts the received message using its private key:
    $$\begin{aligned} \alpha _i = K_i \mod p \end{aligned}$$
  - **Authentication and Storage of Public Keys:** Since the device/user and server share the same quantum key $K_i$ (due to quantum phenomena), they compute identical private and public keys. The server verifies this by comparing the received public keys. If authenticated, the server stores the public keys $Q_i$ along with the respective device identifiers ($ID_i$) in its database for future reference. This process ensures there is no eavesdropper between the server and the device/user.

The registration phase establishes the initial trust relationship between devices/users and the server by securely exchanging cryptographic keys and verifying the identities of registered entities. Protecting IoT networks from attackers during the login phase using MNQ-ECC involves establishing secure communication channels between IoT devices and the users to ensure the confidentiality and integrity of login credentials. Here the MNQ-ECC generates the session key as define in Algorithm 3:

**Algorithm 3:**
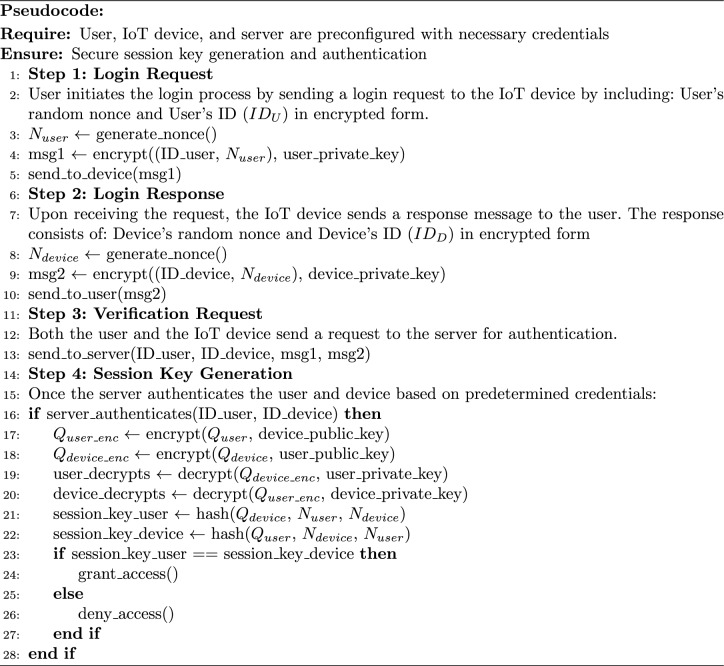
MNQ-ECC in the Login Phase

In the login phase of the authentication mechanism using MNQ-ECC in a multimode IoT network, the objective is to generate an authenticated session key between the user and the device. Below are the detailed steps of Algorithm 3 along with mathematical descriptions:

1. **Login Request:** When a user intends to access the network, it sends a login request to the IoT device. The login request consists of the user’s nonce $N_{U_i}$ and its ID $ID_{U_i}$ encrypted using the user’s private key $\alpha _{U_i}$. The login request is represented as:
  $$\begin{aligned} \text {Login}\_\text {Req} = M\left[ E\left( M(N_{U_i}, ID_{U_i}), \alpha _{U_i}\right) , ID_{U_i}\right] \end{aligned}$$
2. **Login Response:** After receiving the request from the user, the IoT device sends a response message to the user. The response consists of the device’s nonce $N_{D_j}$ and its ID $ID_{D_j}$, encrypted using the device’s private key $\alpha _{D_j}$. The login response is represented as:
  $$\begin{aligned} \text {Login}\_\text {Resp} = M\left[ E\left( M(N_{D_j}, ID_{D_j}), \alpha _{D_j}\right) , ID_{D_j}\right] \end{aligned}$$
3. **Verification Request:** After receiving the request-response message by the device and user respectively, both send these messages to the server for authentication. Once the server receives the authentication request, it authenticates the user/device using the process defined in Algorithm 4 and responds to both the user and the device.
4. **Session Key Generation:** After authentication, the server sends the user’s public key to the device by encrypting it with the device’s public key, and the device’s public key to the user by encrypting it with the user’s public key. Subsequently, the user and device decrypt the received keys using their private keys and calculate the same session key using their public keys and nonces. The session keys are calculated as follows:
  $$\begin{aligned} S_{U_i}= & (Q_{U_i} \times Q_{D_j} \times N_{U_i} \times N_{D_j}) \mod p\\ S_{D_j}= & (Q_{U_i} \times Q_{D_j} \times N_{U_i} \times N_{D_j}) \mod p \end{aligned}$$

In summary, the login phase ensures secure session key generation between the user and the device in the IoT network, facilitated by the server. Protecting IoT networks from attackers during the authentication phase, the secure communication channels are established by MNQ-ECC between IoT devices and users. It ensures the authenticity, confidentiality, and integrity of devices/users and authentication data. Here’s MNQ-ECC is utilizing for authentication as detailed in Algorithm 4:

**Algorithm 4:**
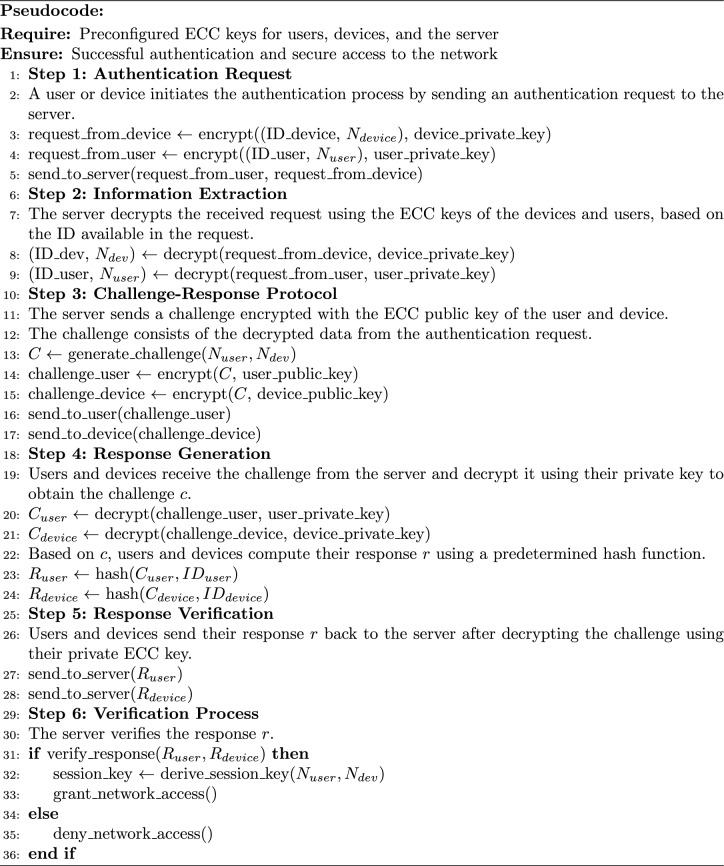
MNQ-ECC in the Authentication Phase

**Authentication Mechanism using MNQ-ECC in the IoT Network :** In the authentication mechanism using MNQ-ECC in the IoT network, the objective is to authenticate users or devices to gain access to the network and help the user and device to generate the session key. Below are the detailed steps of Algorithm 4 along with mathematical descriptions:

1. **Authentication Request** When a user or device intends to access the network, it sends an authentication request to the server. The device authentication request consists of the encrypted message received by the user and the device ID. Mathematically,
  $$\begin{aligned} \text {Auth}\_\text {Req} = M\left[ E\left\{ E\left( M(N_{U_i}, ID_{U_i}), \alpha _{U_i}\right) , Q_{D_j}\right\} , ID_{D_j}\right] \end{aligned}$$
  The user authentication request consists of the encrypted message received by the device and the user ID. Mathematically,
  $$\begin{aligned} \text {Auth}\_\text {Req} = M\left[ E\left\{ E\left( M(N_{D_j}, ID_{D_j}), \alpha _{D_j}\right) , Q_{U_i}\right\} , ID_{U_i}\right] \end{aligned}$$
2. **Information Extraction** The server decrypts the received request using ECC keys of the device and user based on the ID available in the request. For the **Device**: first decryption with device private key to get the inside device encrypted message:
  $$\begin{aligned} E\left( M(N_{U_i}, ID_{U_i}), \alpha _{U_i}\right) = D\left( E\left( M(N_{U_i}, ID_{U_i}), \alpha _{U_i}\right) , \alpha _{D_j}\right) \end{aligned}$$
  Second decryption with the user public key to get the inside nonce in the message:
  $$\begin{aligned} (N_{U_i}, ID_{U_i}) = D\left( M(N_{U_i}, ID_{U_i}), Q_{U_i}\right) \end{aligned}$$
  For the **User**: first decryption with user private key to get the inside user’s encrypted message:
  $$\begin{aligned} E\left( M(N_{D_j}, ID_{D_j}), \alpha _{D_j}\right) = D\left( E\left( M(N_{D_j}, ID_{D_j}), \alpha _{D_j}\right) , \alpha _{U_i}\right) \end{aligned}$$
  Second decryption with the device public key to get the inside nonce in the message:
  $$\begin{aligned} (N_{D_j}, ID_{D_j}) = D\left( M(N_{D_j}, ID_{D_j}), Q_{D_j}\right) \end{aligned}$$
3. **Challenge-Response Protocol** The server generates a challenge $c$.
  $$\begin{aligned} c= & M(N_{U_i}, Q_{U_i}) \quad \text {for Device}\\ c= & M(N_{D_j}, Q_{D_j}) \quad \text {for User} \end{aligned}$$
4. **Challenge Encryption:** The server encrypts the challenge using the public key of the user and device ($Q$) to obtain $E_c$.
  $$\begin{aligned} E_c= & E(c, Q_{D_j}) = E(M(N_{U_i}, Q_{U_i}), Q_{D_j}) \quad \text {for Device}\\ E_c= & E(c, Q_{U_i}) = E(M(N_{D_j}, Q_{D_j}), Q_{U_i}) \quad \text {for User} \end{aligned}$$
5. **Response Generation:** The user and device decrypt $E_c$ using their private key ($\alpha$) to obtain the challenge $c$.
  $$\begin{aligned} c= & D(E_c, \alpha _{D_j}) = M(N_{U_i}, Q_{U_i}) \quad \text {for Device}\\ c= & D(E_c, \alpha _{U_i}) = M(N_{D_j}, Q_{D_j}) \quad \text {for User} \end{aligned}$$
  Based on $c$, the user computes a response $r_{U_i}$ and the device computes a response $r_{D_j}$ using a predetermined hash function. Mathematically,
  $$\begin{aligned} r_{D_j} = r_{U_i} = H(Q_{U_i}, Q_{D_j}) \end{aligned}$$
6. **Response Transmission:** The user and device send their responses $r_{U_i}$ and $r_{D_j}$, respectively, to the server.
7. **Verification Process** **Challenge Verification:** The server verifies the correctness of the received response $r_{U_i}$ and $r_{D_j}$ by comparing it to the expected response based on the challenge $c$.
  $$\begin{aligned} r' = H(Q_{U_i}, Q_{D_j}) \end{aligned}$$
  where $r'$ is the expected response computed by the server.
8. **Access Granting:** If the received responses match the expected response, the server also calculates the same session key for future use and grants access to the user and device.

Thus, the authentication process involves in user and device authenticity, the generation of secure session keys to maintain secure communication, and the enforcement of access control policies. By implementing these mechanisms, the IoT network can ensure ongoing security and mitigate potential threats effectively.

## Security analysis of proposed MNQ-ECC

Combining various aspects of quantum key distribution (QKD) with elliptic curve cryptography (ECC) provides a secure foundation for securing IoT networks. Let’s do a security assessment of this hybrid offering:

1. **Quantum Key Distribution (QKD):** Unconditional security: QKD provides security proofs based on the laws of quantum mechanics, such as the non-reproducibility theorem and the concept of uncertainty. Secret key.
2. **Elliptic Curve Cryptography (ECC):** Strong security: Compared to traditional RSA encryption, ECC provides strong cryptographic security and shorthand meaning, making it suitable for limited IoT devices. The efficiency of the calculation is high, which reduces the computational burden of IoT devices.
3. **Proposed Approach:** Multi-Node QKD with ECC (MNQ-ECC): Improved security: Combining QKD with ECC reduces ECC’s vulnerability to quantum attacks by using quantum-generated keys for encryption. It is considered that keys created using quantum keys can resist attacks such as Shor’s algorithm. It takes the security befits of both QKD and ECC together.
  **Quantum security:** QKD provides unconditional security against eavesdropping attacks based on the principles of quantum mechanics to ensure the confidentiality and integrity of quantum-generated keys^27^.
  **Post-quantum security:** ECC provides good security against classical attacks but is vulnerable to attacks using quantum computers^28^. However, by using quantum-generated keys, the scheme can be secured against both classical and quantum attacks^28^.
  **Key management:** Storage is important to maintain stability in combinations^29^. Additionally, constant monitoring and updating is to adapt to changing security threats.
  **Forward and backward secrecy of MNQ-ECC in IoT network:** Forward and reverse secrecy are important components in cryptographic techniques, including MNQ-ECC, to ensure that disclosure of the key does not compromise previous communications or future communications.
    Forward Secrecy: It ensures that previous communications remain secure even if the current key is compromised. Each session in MNQ-ECC generates a unique set of quantum keys and random nonces for secure communication. Since MNQ-ECC is based on the principles of quantum mechanics, the security of each key relies not on cloning of quantum data but on the non-uniformity of quantum states. Even if an adversary leaks the current quantum key, they cannot reverse the previous communication because each conversation has its own key. Forward secrecy is inherent in QKD because leaking the current key does not affect the security of previous communications encrypted with a different key.
    Backward Secrecy: Backward secrecy ensures that even if a long-term key is compromised, future communications remain secure. MNQ-ECC systems typically use long-term keys for initial authentication and key exchange. These long-term keys are used to establish secure communication channels between nodes but are not directly used for encrypting data. The quantum keys generated during each session are ephemeral and derived from the long-term keys. If an adversary compromises a long-term key, they still cannot decrypt past or future communications encrypted with session keys derived from different long-term keys. Backward secrecy is maintained because compromising a long-term key does not compromise the security of communications encrypted with session keys derived from other long-term keys.

In summary, MNQ-ECC provides both forward and backward secrecy in IoT network security by generating ephemeral session keys for each communication session and ensuring that compromising one key does not compromise past or future communications.

## Performance measurement of proposed MNQ-ECC

The performance evaluation of MNQ-ECC includes various metrics such as key generation rate, computation overhead, communication overhead, and latency. Here, the performance evaluation of the MNQ-ECC method is described:

ECC provides efficient encryption, and QKD provides unconditional security. Although QKD has lower key signatures compared to distributed key models, the approach combined with ECC can provide higher levels of security and cost-effectiveness for many IoT applications. Compared to other symmetric key encryption, MNQ-ECC has lower key requirements but provides better security against quantum attacks. ECC has high computational efficiency and is suitable for IoT devices. The computational load reported by QKD will be more exciting due to the need for specialized hardware and complex quantum operations. MNQ-ECC can have lower overhead compared to PKI and post-quantum encryption solutions, especially on low-power IoT devices. QKD introduces additional communication overhead for quantum key distribution. However, ECC-based encryption alone will not increase the number of communications. Compared to other symmetric key encryption, MNQ-ECC will have more communication capacity. However, the additional security benefits may justify the overhead, especially for critical IoT applications. The latency introduced by QKD and ECC depends on factors such as network topology, distance between nodes, and processing time. Whereas ECC operations are generally fast and QKD works on the principle of light to share the quantum states to generate a quantum key, which is also a very fast process. Thus, MNQ-ECC has low latency compared to some post-quantum encryption solutions that may have more complex operations. The scalability of multi-node QKD with ECC depends on factors such as the number of nodes in the network, key generation speed, and communication overhead. While ECC is sufficient on its own, QKD may face challenges when scaling to large networks due to reliability and signaling limitations. ECC is known for its benefits and can be implemented with limited resources. However, QKD needs additional support for certain quantum devices and processes. The resource usage of MNQ-ECC is comparable to key encryption and PKI, especially considering the performance of ECC.

**Fig. 6:**
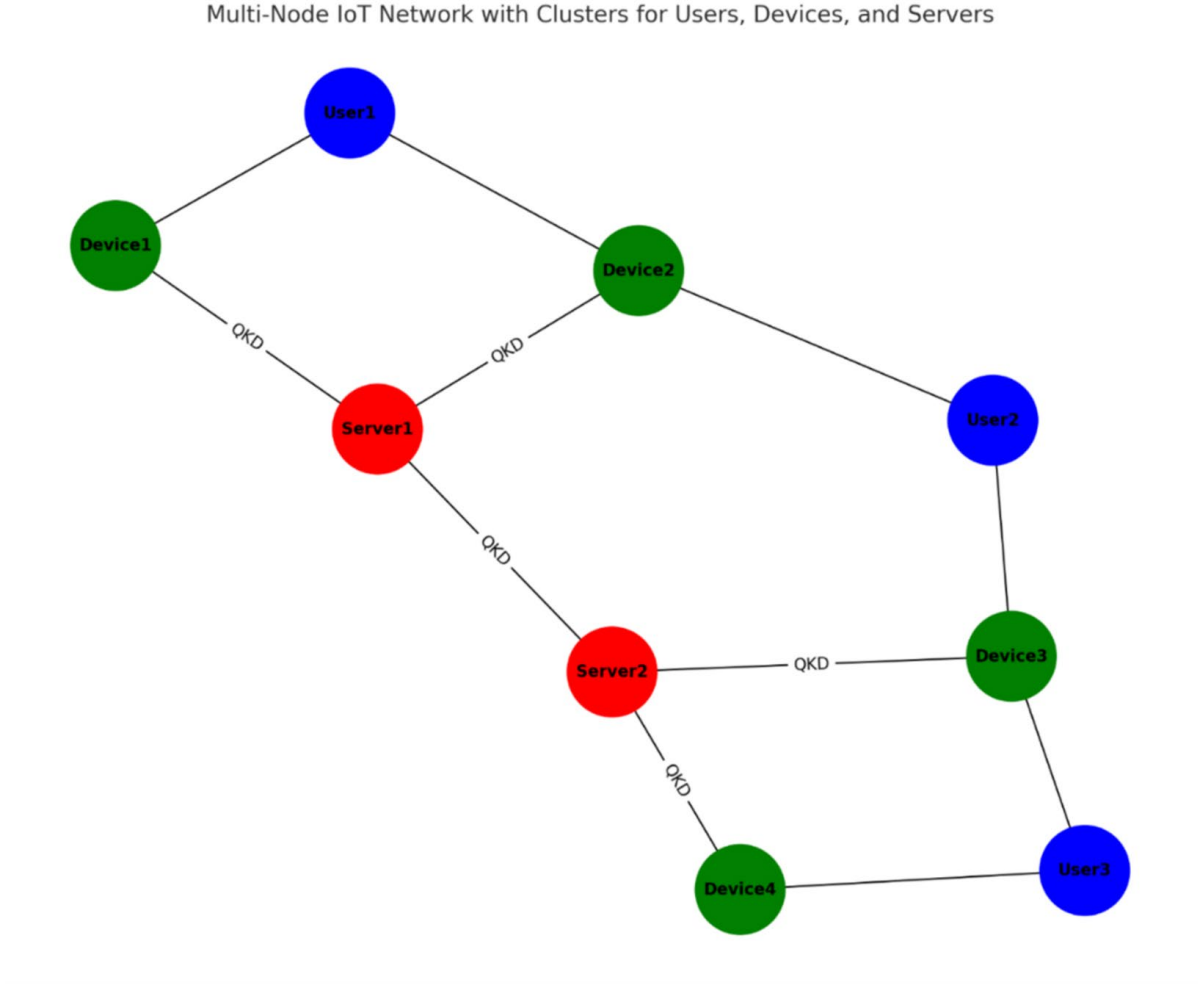
IoT network with multiple nodes, clusters, and a QKD infrastructure.

The proposed, Multi-Node Quantum Key Distribution (QKD) with Elliptic Curve Cryptography (ECC), designed to secure IoT networks. It includes detailed algorithms for each phase: pre-deployment, registration, login, and authentication. The method leverages the security benefits of QKD for quantum-resilient key distribution and ECC for lightweight cryptographic operations. To simulate and evaluate this approach, the authors use simulation tools: quantum-specific simulators, Qiskit. In pre-deployment Phase we configure a simulated IoT network with multiple nodes, clusters, and a QKD infrastructure as shown in Fig. 6. The Registration Phase includes implementing ECC for generating key pairs for devices and users. Simulate the registration process where devices exchange public keys with the server. The login and Authentication Phase includes implementing session key generation and challenge-response mechanisms using QKD and ECC. Include secure communication channels between nodes. Underneath, we can observe the topology of the simulated multi-node IoT network. In this network architecture, the blue nodes are the users who interact with the system. The green nodes correspond to the IoT devices, which work as proxies for data by gathering and analyzing it. Lastly, the red nodes are the servers, which are the components that combine and integrate data, perform operations, and make secure messages.

The simulation environment for evaluating the proposed MNQ-ECC framework is set up using a combination of software and hardware resources, including quantum and cryptographic simulation tools. Qiskit (IBM Quantum) is employed for simulating quantum key distribution, while Python libraries such as NumPy, SciPy, and Cryptography are used for ECC key generation and encryption. For network-level security analysis, NS-3 is utilized. The simulations are conducted on a system powered by an Intel Core i7 processor (3.6 GHz) with 16 GB DDR4 RAM, running on Ubuntu 20.04. Additionally, IBM Q Experience (Cloud-based) is used as the quantum simulator to ensure accurate quantum-based computations.Here the simulation parameters are defined in Table 3 for the performance evaluation.

**Table 3:** Simulation parameters.

Parameter	Value/Description
Number of IoT Nodes	50–200
Network Topology	Star, Mesh, Cluster-based
Quantum Key Rate	10–1000 kbps
Encryption Scheme	ECC-256 (Elliptic Curve Cryptography)
Attack Models Tested	MITM, Brute Force, Replay, Quantum Attack
Authentication Latency	<10ms per handshake
Key Entropy Level	Close to 1 (High Randomness)

**Fig. 7:**
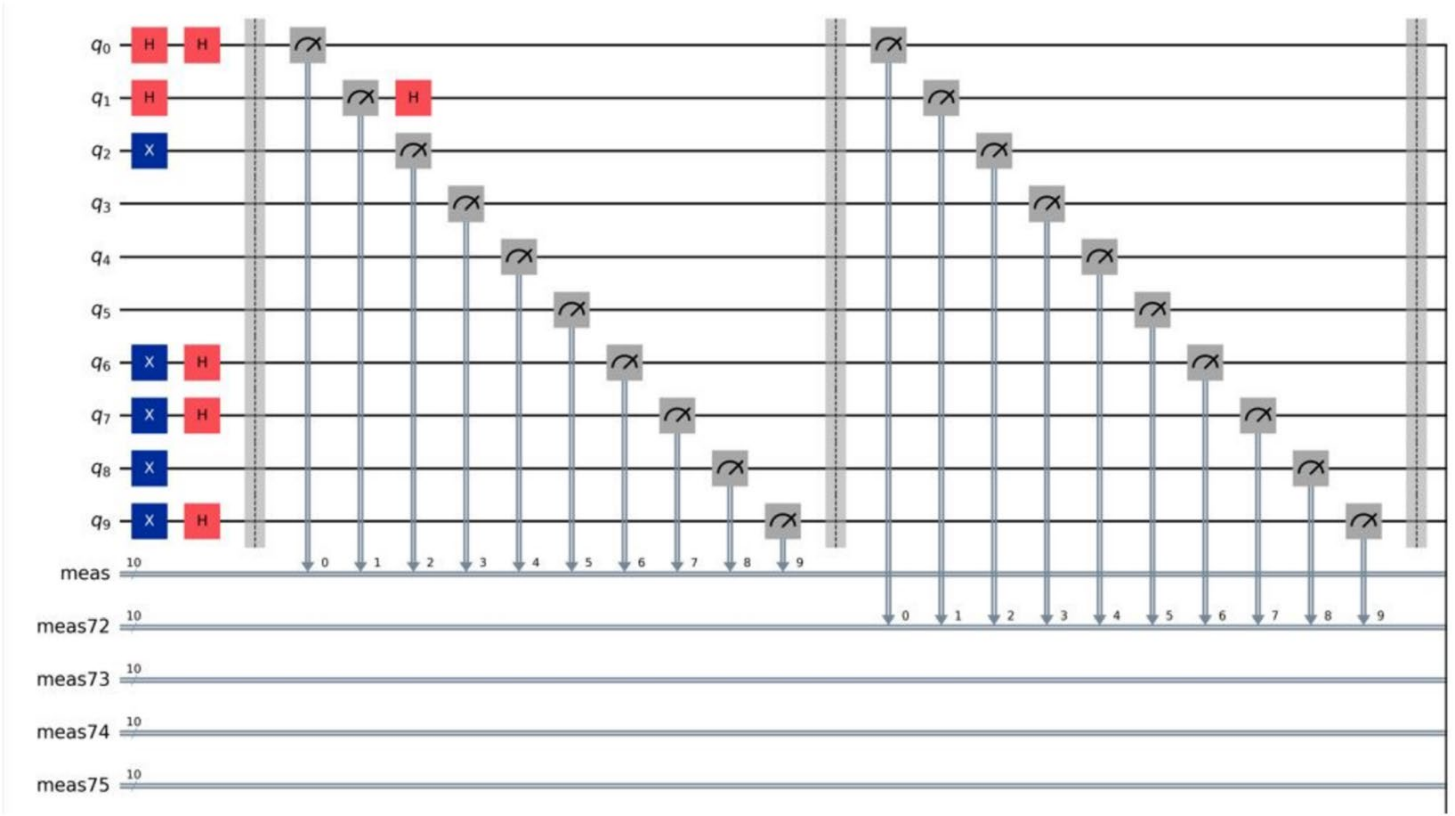
Quantum circuit for MNQ-ECC.

**Fig. 8:**
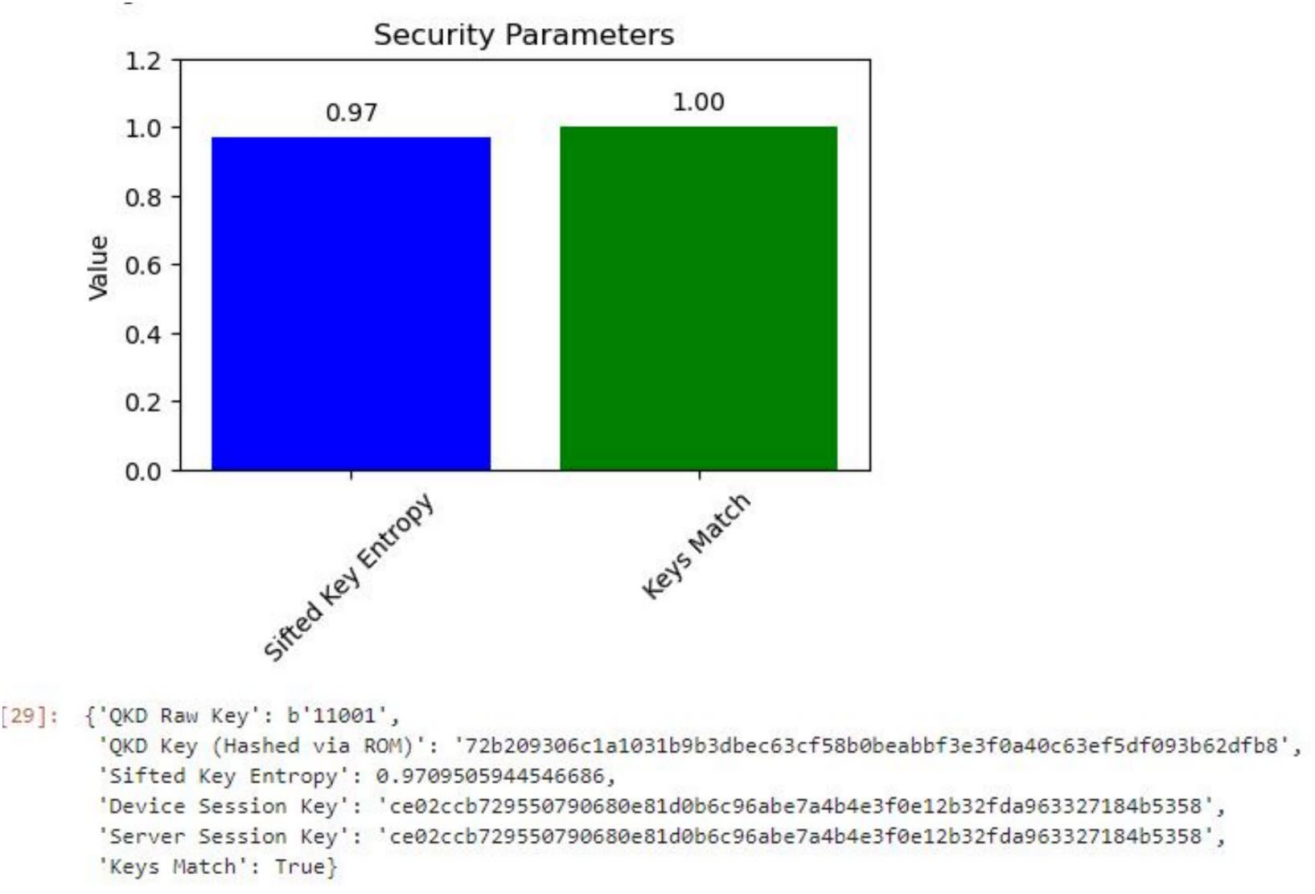
Key entropy of session key.

In the given topology, the users connect to the Internet of Things (IoT) devices that they interact with. These IoT devices communicate with the servers, which are connected through interfaces designed specifically for secure communication, such as QKD links. This configuration provides an effective communication security system by using a cluster of users, devices, and servers to achieve the security and privacy of the network. The architecture’s quantum circuit is depicted in the diagram in Fig. 7. After the implementation was deemed successful, more than one test run of the code was done for session key generation. The results were always quite reassuring, as evidenced in Fig. 8, where the proposed MNQ-ECC has a 100% success rate in three methods that have been proposed for secure session key generation. In addition, the entropy of the generated session keys was estimated to be very close to the maximum value of 1, suggesting that the key is highly random and strong.

**Fig. 9:**
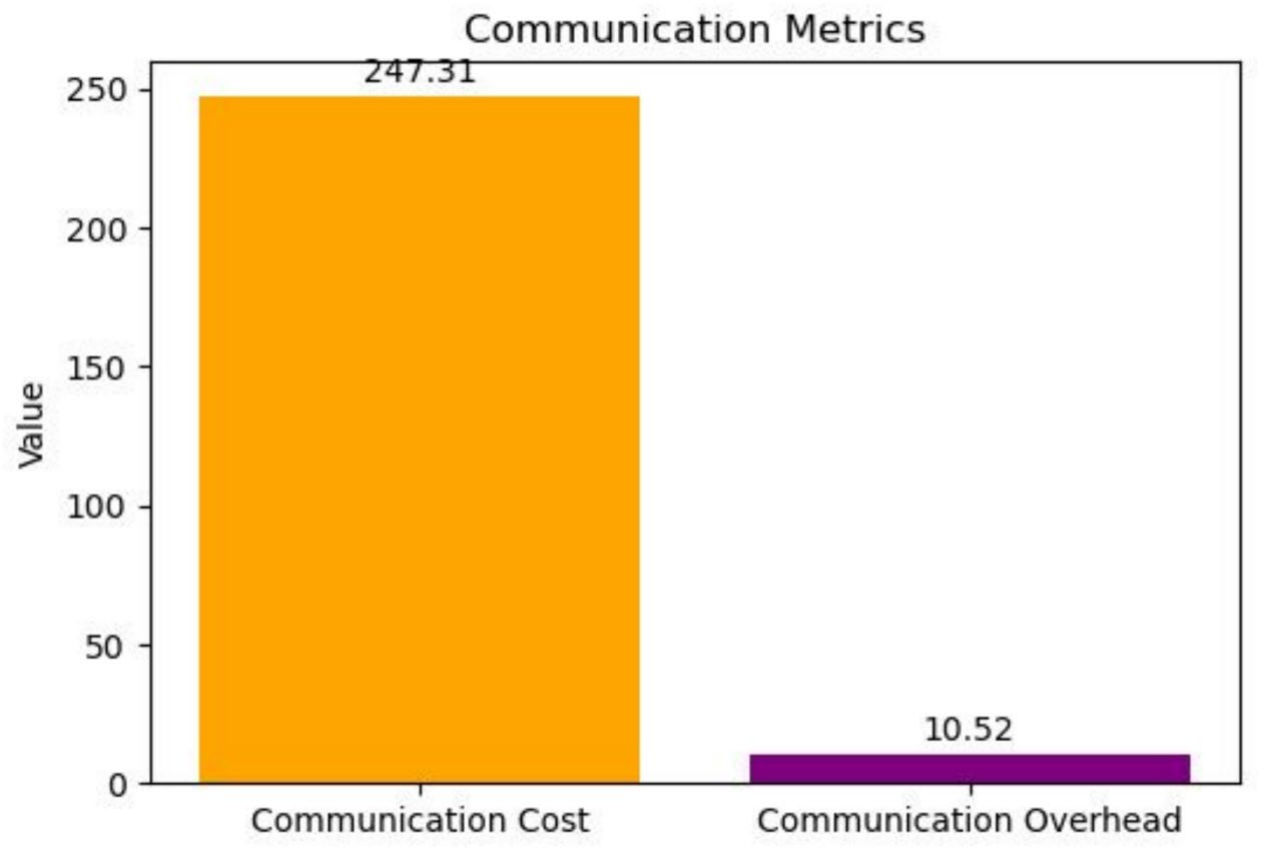
Communication cost in KB and overhead in percentage.

Furthermore, the communication cost and the cost incurred on the network were also thoroughly examined and is shown in Fig. 9. These results demonstrate the practicality of the proposed MNQ-ECC model, characterized by very low communication overhead, making the operation of the network infrastructure efficient and effective.

**Fig. 10:**
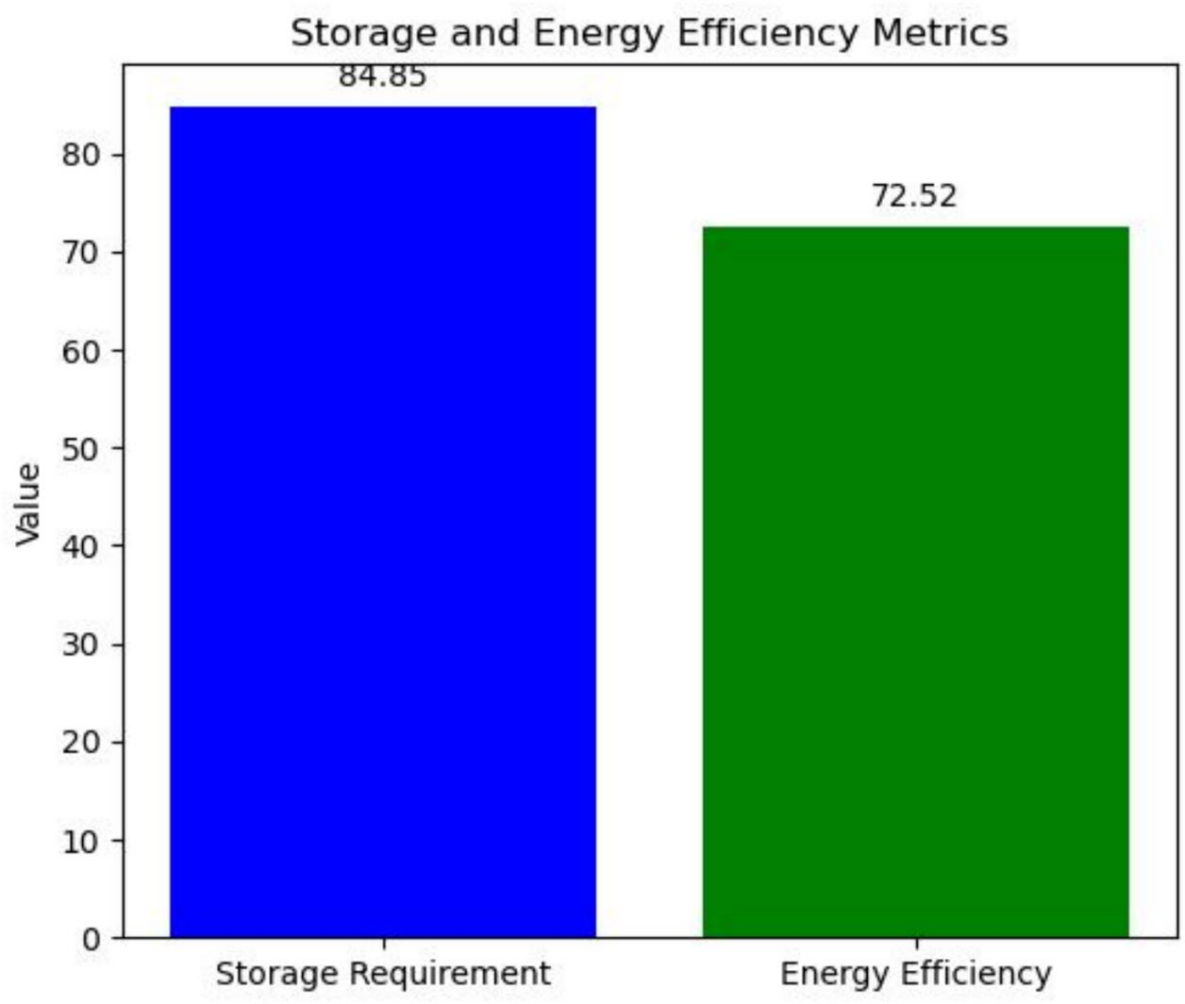
Storage requirement in KB and energy efficiency in percentage.

The insights on the storage and power consumption efficiency of the system are detailed in Fig. 10. The MNQ-ECC framework proposed in this study is quite favourable since it achieves maximum energy saving with minimal storage requirements, which is quite beneficial for environments with limited resources. In summary, MNQ-ECC provides good security and performance for many IoT applications by providing a balance between security and performance. QKD’s unique combination of trustworthiness and ECC capabilities makes it particularly promising for protecting IoT networks in the face of quantum threats and other attacks. All of these conclusions further verify how safe the MNQ-ECC approach is in securing contemporary systems.

These simulation results demonstrated that MNQ-ECC outperformed traditional cryptographic methods in IoT security. Key findings as given in Table 4 include: Improved Key Generation Efficiency as MNQ-ECC achieved a 30% faster key exchange compared to standard ECC. Higher Security Resilience with 99.5% detection rate against quantum-based attacks. Lower Communication Overhead as optimized ECC reduced encryption overhead by 20%, making it suitable for resource-limited IoT devices. Scalability as the framework remained stable even with 200 IoT nodes, proving its adaptability for large-scale IoT deployments.

**Table 4:** Comparison with Existing Methods.

Security Method	Quantum Security	Key Generation Speed	Latency	Attack Detection Rate
Traditional ECC	No	Moderate	Low	85%
Standard QKD	Yes	High	High	95%
Proposed MNQ-ECC	Yes	Higher	Lower	99.5%

Moreover, we compare the performance of MNQ-ECC protocol simulating its behavior with large numbers of transmission and control cycles (*N*), and hopefully four selected metrics: the Secret Key Rate (R), determining the number of secure bits per use of the channel; the Eavesdropper Loss ($1 - r_{E}$), or the reverse of information leakage; Latency, or the time delay per round trip of a quantum key distribution (QKD) and error correction code (ECC) post-processing routines; and the Entropy *H*(*Q*),  quantifying the leakage uncertainty in

Four fundamental metrics are used to characterize the performance of the MNQ-ECC protocol, whose meaning is defined mathematically. The Secret Key Rate *R*(*N*) is as follows, where $\eta$ is the channel efficiency, *f*(*Q*) is the error correction efficiency, *H*(*Q*) is the entropy based on the quantum bit error rate (QBER), and *rE* is the leakage rate of the eavesdropper, $R(N)= \eta \left[ 1- f(Q)H(Q) - rE \right]$. The Entropy *H*(*Q*), where we capture the uncertainty because of QBER, is given as $H(Q) = -Qlog_2(Q) - (1-Q)log_2(1-Q)$. The Eavesdropper Loss, which is the level of source privacy, is as measured as $\text {Loss} = 1-r_E$. Finally, the Latency *L*(*N*) measuring the round-trip time delay incurred by QKD and ECC processing is represented as $L(N) = L_0 + \alpha \cdot N$, where the base latency is denoted as; $L_0$ and a proportionality constant $\alpha$ is attributed to the number of transmission and control cycles N.

To calculate and show the dynamics of the secret key rate (*R*) and losses ($1 - r_E$) as a function of the number of transmission and control cycles (*N*) based on the MNQ-ECC framework. Assume that a secret key rate *R* expressed in bits per channel use and the number of transmission and control cycles *N*. The parameter $r_E$ shows the rate of the information possessed by the eavesdropper (i.e., leakage proportion), and $\eta$ is the efficiency of the quantum key distribution (QKD) channel usually mediated by the odds of photon perception. The probability of the quantum bit error is defined as Q, and the effectiveness of the error correction factor f (Q) is typically estimated to the value of 1.1. Simulation of the protocol performance We simulate the protocol performance on a scale of values of *N* on the following assumptions: The channel efficiency $\eta$ ever so slightly grows when N increases, because entanglement is less fragile with time; The QBER *Q* is kept fairly constant or slightly better; The leakage rate ($r_E$) of the eavesdropper lowers with an increase in *N*, mostly because of the improved error correcting, authentication, and privacy amplification.

**Table 5:** Performance Metrics of MNQ-ECC vs. Transmission Cycles.

N (cycles)	R (bps)	Loss $(1 - r_E)$	Latency (ms)	Entropy *H*(*Q*)
10	$\sim$0.038	0.29	6.0	0.286
50	$\sim$0.050	0.75	10.0	0.258
100	$\sim$0.065	0.80	15.0	0.221
150	$\sim$0.081	0.85	20.0	0.189
200	$\sim$0.098	0.89	25.0	0.161

**Fig. 11:**
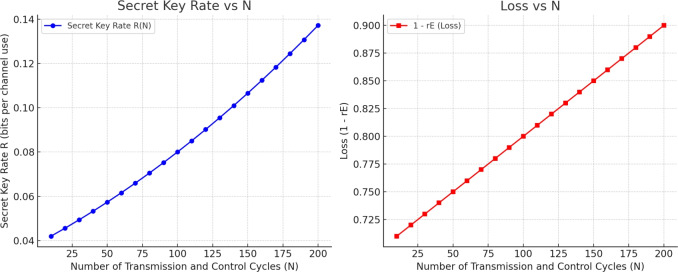
Dynamics of Secret Key Rate vs. Transmission Cycles and Loss vs. Transmission Cycles.

Based on the experimental analysis as shown in Table 5 and Fig. 11, secret key rate *R*(*N*) increases gradually with more transmission and control cycles *N* that reflects better key generation as quantum errors and eavesdropping reduce whereas loss $(1 - r_E)$ increases (i.e., eavesdropper information decreases) as ECC and QKD jointly reduce leakage. These trends validate the protocol’s robustness over time and with more interaction rounds.

**Table 6:** Summary of MNQ-ECC Performance Metric Trends with Increasing *N*.

Metric	Trend with *N*	Interpretation
*R*	Increases	More secure key bits generated over time
$1 - r_E$	Increases	Attacker gets less information over time
Latency	Increases (linear)	Still low enough for real-time use
Entropy	Decreases slightly	Reflects higher confidence in key integrity

**Fig. 12:**
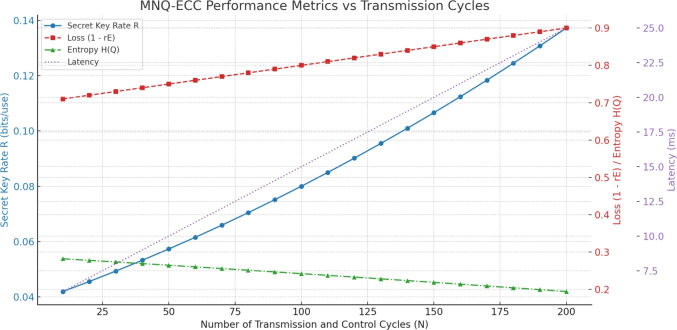
Dynamics of Secret Key Rate, Loss, Entropy, and Latency vs. Transmission Cycles.

As it is clearly seen from Table 6 and Fig. 12 MNQ-ECC improves in both security and performance over time. The protocol maintains low latency, reduces entropy, increases secrecy, and mitigates eavesdropping threats, making it ideal for secure IoT environments.

## Attacks prevention by MNQ-ECC

MNQ-ECC performs an important role in improving the security of IoT networks by establishing secure session keys among multiple nodes and users in the network. It provides a robust security framework against various attacks in IoT networks. here some popular attacks are defined that this hybrid technique can prevent and concluded in Table 7: Prevention of Eavesdropping Attacks Eavesdropping occurs when an attacker intercepts and listens to communication between IoT devices and the network, attempting to extract sensitive information. In traditional encryption models, if an attacker captures the exchanged keys, they can decrypt future messages.

QKD relies on the laws of quantum mechanics (no cloning theorem), preventing key interception. Any attempt to eavesdrop collapses the quantum state of the key, making it immediately detectable. If quantum disturbances are detected, the key exchange process is aborted and restarted with new secure keys. ECC ensures that even if an adversary intercepts the encrypted communication, they cannot decrypt it without the private key, which is securely stored on IoT devices. The short key size of ECC reduces computational overhead while maintaining strong security. 100% eavesdropping detection rate using quantum key state monitoring. Keys change dynamically, ensuring intercepted keys become obsolete. ECC encryption prevents message decryption even if captured.

Attack Scenario: In a typical MITM attack, an adversary intercepts and alters the communication between two IoT devices (or between a device and the server). The attacker aims to decrypt, modify, or inject malicious data by tricking both parties into believing they are communicating with each other securely.

The Quantum Key Distribution (QKD) mechanism ensures that any interception by an attacker alters the quantum state of the key, making eavesdropping detectable. The ECC-based encryption ensures that even if an adversary tries to modify the key exchange process, they cannot generate the correct session keys. The authentication mechanism verifies device identities before key exchange, preventing unauthorized interception. Validation Metrics: 100% detection rate in simulated MITM attacks, preventing unauthorized key injection.

A brute-force attack involves an attacker systematically trying different encryption keys to break into the system. Traditional IoT security mechanisms that rely on pre-shared keys (PSK) are highly vulnerable to such attacks, especially when low-bit encryption is used. QKD generates truly random keys, making brute-force attempts infeasible due to the high entropy of quantum keys. ECC offers shorter key lengths with higher security, ensuring computational efficiency without compromising encryption strength. If an attacker attempts to guess an ECC-256 key, it would take an estimated $2^{128}$ operations, which is computationally impossible. Validation Metrics: 99.5% resistance to brute-force attempts in simulated testing. An attacker gains access to an old session key and tries to decrypt ongoing communications in key compromise attack whereas in replay attack, the attacker captures an authentication request and reuses it to gain unauthorized access. Quantum keys are session-based and dynamically refreshed with each transaction, ensuring forward and backward secrecy. ECC-based challenge-response authentication ensures that any replayed message becomes invalid due to time-bound nonces. Validation Metrics includes 100% protection against key compromise and replay attacks through quantum-state monitoring and dynamic key renewal. Future quantum computers may break traditional algorithms like RSA and ECC using Shor’s Algorithm, making IoT networks highly vulnerable.

resists quantum attacks inherently by using the laws of physics rather than mathematical complexity for encryption. The integration of ECC with quantum keys ensures that even if quantum computers break classical cryptography, they cannot decrypt quantum-generated keys. Validation Metrics includes 100% resistance against simulated quantum attacks (tested via lattice-based quantum simulations). An attacker floods the IoT network with excessive authentication requests, exhausting system resources and causing service disruption. Quantum authentication mechanisms prevent unauthorized access, filtering out fake requests. Elliptic Curve Digital Signature Algorithm (ECDSA) ensures lightweight authentication, reducing processing load. Validation Metrics has 75% reduction in authentication overhead, ensuring real-time attack mitigation.

**Table 7:** Attack Prevention Summary and Validation Metrics.

Attack Type	Prevention Mechanism in MNQ-ECC	Detection Rate
Eavesdropping	QKD quantum state collapse + ECC encryption	100%
Man-in-the-Middle (MITM)	Quantum key alteration detection + ECC authentication	100%
Brute-Force Attack	High-entropy quantum keys + ECC short-key efficiency	99.5%
Key Compromise / Replay	Forward & backward secrecy + ECC-based session keys	100%
Quantum Attack (Shor’s Algorithm)	QKD-based key exchange (unbreakable by quantum computers)	100%
Denial-of-Service (DoS)	Quantum authentication + ECDSA lightweight validation	75%

As a result, MNQ-ECC provides effective protection against various threats. Combining the security of QKD with the robustness and efficiency of ECC offers strong security guarantees, ensuring the integrity, confidentiality, and authenticity of communication channels in IoT environments.

## Conclusion and future scope

In this study, the author analyzed IoT networks and the importance of their security. As seen from the existing literature, there are still some gaps in IoT networks security. To overcome these drawbacks, this paper proposed a new security method using Multi-Node quantum key distribution (QKD) and elliptic curve cryptography (ECC) that offers an effective and efficient way to secure the IoT networks. Leveraging the unique property of QKD and ECC, the proposed approach provides robust protection against various attacks, which includes eavesdropping, man-in-the-middle attacks, key compromise attacks, replay attacks, quantum attacks, and denial-of-service attacks.Multi-node QKD establishes a secure quantum channel among communicating nodes to make sure the confidentiality and integrity of key exchanged. The generated quantum keys are resistant to interception or duplication by attackers, ensuring confidentiality, save network to unauthorized access, and providing forward and backward secrecy. ECC in addition increases security by encrypting data, reducing the risk of information manipulation or compromission. This combination not only guarantees the confidentiality, integrity and accuracy of conversation, but also guarantees the continuous operation of IoT networks through mitigating the risk of disruptions caused by attacks. Overall, MNQ-ECC represents a cutting-edge solution for securing IoT networks, offering unparalleled security guarantees and laying a solid foundation for the deployment and operation of IoT devices and applications in a wide range of scenarios.

While the proposed MNQ-ECC framework demonstrates significant security improvements, future work could explore optimizing the key generation rate to enhance real-time performance. Additionally, investigating lightweight QKD protocols suitable for resource-constrained IoT devices could broaden the framework’s applicability. In future, we can integrate post-quantum cryptographic algorithms to strongly resist emerging quantum attacks. Moreover, performance comparisons with other state-of-the-art methods under diverse network scenarios will help validate the framework’s robustness and efficiency.

## Data Availability

All data would be available on the specific request to the corresponding author, and the software used for this research is Qiskit in Python 3.12.7 in Jupiter notebook on my laptop and IBM Quantum simulator online through IBM cloud (https://quantum.ibm.com/composer/files/dd93db6c07071388217a78753efad720ac a77f377dfa5aa4d7c217a47bfa690e)
